# 1-(Benzo[*d*]thiazol-2-yl)-3-phenylureas as dual inhibitors of casein kinase 1 and ABAD enzymes for treatment of neurodegenerative disorders

**DOI:** 10.1080/14756366.2018.1445736

**Published:** 2018-03-14

**Authors:** Ondrej Benek, Lukas Hroch, Laura Aitken, Frank Gunn-Moore, Lucie Vinklarova, Kamil Kuca, Daniel I. Perez, Concepcion Perez, Ana Martinez, Zdenek Fisar, Kamil Musilek

**Affiliations:** a University of Hradec Kralove, Faculty of Science, Department of Chemistry, Hradec Kralove, Czech Republic;; b University Hospital in Hradec Kralove, Biomedical Research Center, Hradec Kralove, Czech Republic;; c National Institute of Mental Health, Klecany, Czech Republic;; d University of St. Andrews, Medical and Biological Sciences Building, School of Biology, St. Andrews, UK;; e Centro de Investigaciones Biologicas-CSIC, Madrid, Spain;; f Instituto de Quimica Medica-CSIC, Madrid, Spain;; g Charles University and General University Hospital in Prague, First Faculty of Medicine, Department of Psychiatry, Prague, Czech Republic

**Keywords:** Alzheimer’s disease, amyloid-beta binding alcohol dehydrogenase (ABAD), benzothiazole, casein kinase 1 (CK1), neurodegeneration

## Abstract

Several neurodegenerative disorders including Alzheimer’s disease (AD) have been connected with deregulation of casein kinase 1 (CK1) activity. Inhibition of CK1 therefore presents a potential therapeutic strategy against such pathologies. Recently, novel class of CK1-specific inhibitors with *N*-(benzo[*d*]thiazol-2-yl)-2-phenylacetamide structural scaffold has been discovered. 1-(benzo[*d*]thiazol-2-yl)-3-phenylureas, on the other hand, are known inhibitors amyloid-beta binding alcohol dehydrogenase (ABAD), an enzyme also involved in pathophysiology of AD. Based on their tight structural similarity, we decided to evaluate series of previously published benzothiazolylphenylureas, originally designed as ABAD inhibitors, for their inhibitory activity towards CK1. Several compounds were found to be submicromolar CK1 inhibitors. Moreover, two compounds were found to inhibit both, ABAD and CK1. Such dual-activity could be of advantage for AD treatment, as it would simultaneously target two distinct pathological processes involved in disease’s progression. Based on PAMPA testing both compounds were suggested to permeate the blood-brain barrier, which makes them, together with their unique dual activity, interesting lead compounds for further development.

## Graphical abstract

**Figure:**
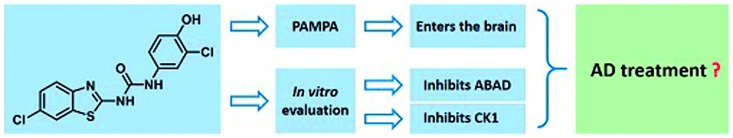

## Introduction

Enzyme casein kinase 1 (CK1), a member of a Ser/Thr specific protein kinase superfamily, is ubiquitously expressed in eukaryotic organisms. At least seven CK1 isoforms (α, β, γ1–3, δ, and ε) and their various splice variants have been currently identified in mammals^1^. Aberrant functional regulation of CK1, such as its over-expression or excessive activation, is implicated in the pathogenesis of many diseases including several neurodegenerative disorders, namely Alzheimer’s disease (AD), Parkinson’s disease (PD), amyotrophic lateral sclerosis (ALS) and frontotemporal lobar degeneration (FTLD)^1^.

In connection to AD, it was suggested that isoform CK1δ (together with GSK3β) be responsible for tau phosphorylation^2^. However, in distribution and co-localization studies CK1δ was rather associated to granulovacuolar degeneration bodies whereas CK1α was observed to co-localize with neurofibrillary lesions^3^. Additionally, isoform CK1ε was found to modulate, either directly or indirectly, the activity of γ-secretase and consequently the production of Aβ. Accordingly, inhibitors of CK1ε were able to reduce Aβ production by γ-secretase, moreover, without affecting Notch cleavage^4^. In PD CK1 seems to participate in phosphorylation of α-synuclein and parkin, both proteins significantly involved in PD pathophysiology^1^. ALS and FTLD are another two neurodegenerative diseases connected with aberrant CK1δ activity, which is responsible for abnormal phosphorylation of diseases associated protein TDP-43^5^.

**Figure 1.**
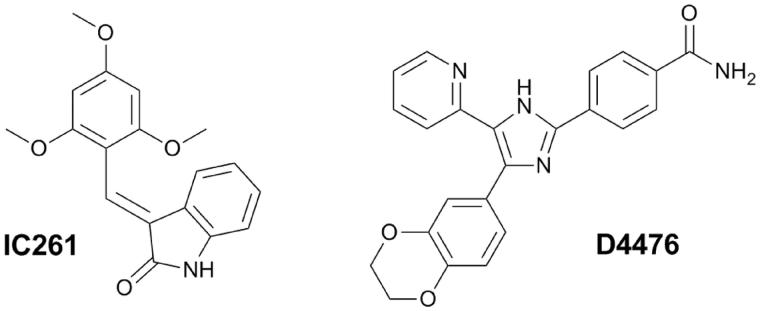
Structures of known CK1-selective inhibitors.

Taken together, CK1 inhibition presents a promising therapeutic strategy for the treatment and prevention of neurodegenerative diseases mentioned above. So far, numerous CK1 inhibitors have been described, which was comprehensively summarized by Perez et al.^1^. Out of the currently known CK1-specific inhibitors, compounds **IC261** and **D4476** were found with the highest selectivity and were characterized within *in vitro* and *in vivo* administration (Figure 1)^1^. However, there is a need for potent and isoform specific CK1 inhibitors with optimized pharmacological profile enabling penetration through blood–brain barrier (BBB).

Recently, a series of *N*-(benzo[*d*]thiazol-2-yl)-2-phenylacetamides have been described as novel class of selective CK1 inhibitors. These compounds with IC_50_ for CK1δ in nanomolar scale were predicted to cross BBB and showed protective effect on *in vivo* hTDP-43 neurotoxicity *Drosophila* model, which makes them potential pharmacological treatment of human TDP-43 proteinopathies such as ALS as well as other neurodegenerative disorders connected with abnormal CK1 activity^6^.

**Figure 2.**
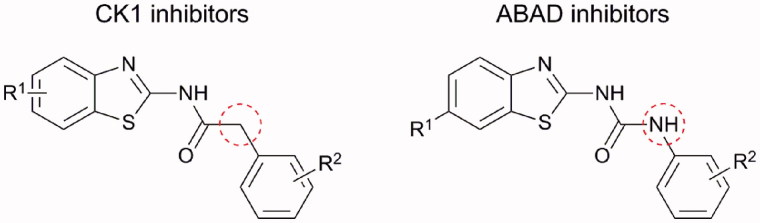
Structural similarity of CK1 and ABAD inhibitors.

Above mentioned *N*-(benzo[*d*]thiazol-2-yl)-2-phenylacetamide CK1 inhibitors are very similar in their structure to some 1-(benzo[*d*]thiazol-2-yl)-3-phenylureas^7^, which were originally prepared and tested as inhibitors of amyloid-beta binding alcohol dehydrogenase (ABAD) for treatment of AD. They both (CK1 and ABAD inhibitors) consist of benzothiazolyl and phenyl moiety connected through the three-membered linker and in both cases, the benzothiazolyl moiety is substituted in position 6 with small electron withdrawing group (Figure 2)^6^ ^,^ ^7^. Therefore, the two structural cores differ only by the “classical” bivalent isosteric replacement of –CH_2_– by –NH– (Figure 2)^8^.

Based upon this structural similarity we decided to evaluate the inhibitory activity of the previously published benzothiazolylphenylureas^7^ against CK1. Isoforms CK1δ and CK1ε were chosen for the testing as they are mainly associated with neurodegenerative disorders. Additionally, compounds inhibiting both, CK1δ/ε and ABAD^9^, would be of further interest due to their simultaneous action against two distinct pathological mechanisms involved in AD and as such would present novel multi-target-directed ligands (MTDLs) different from commonly developed MTDL heterodimers^10^.

## Experimental

### Chemicals

Synthesis and identification of tested compounds was previously published by Hroch et al.^7^

### CK-1δ and CK-1ε assay protocol

The Kinase-Glo Kit was purchased from Promega (Promega Biotech Ibérica, SL) and was used to screen compounds for activity against CK-1δ and CK1ε. Human recombinant Casein Kinase 1 delta and epsilon was purchased from Millipore (Millipore Iberica S.A.U.). Casein solution from bovine milk, 5%, was purchased from Sigma-Aldrich (St. Louis, MO, USA). ATP and all other reagents were from Sigma-Aldrich (St. Louis, MO, USA). Kinase-Glo assays were performed in assay buffer using black 96-well plates. In a typical assay, 10 µL of test compound (dissolved in DMSO at 1 mM concentration and diluted in advance in assay buffer to the desired concentration) 10 µL (16 ng) of enzyme were added to each well followed by 20 µL of assay buffer containing 0.1% casein as substrate and 4 µM ATP. The final DMSO concentration in the reaction mixture did not exceed 1%. After 60 min incubation at 30 °C the enzymatic reaction was stopped with 40 µL of Kinase-Glo reagent. Glow-type luminescence was recorded after 10 min using a FLUOstar Optima (BMG Labtechnologies GmbH, Offenburg, Germany) multimode reader. The activity is proportional to the difference of the total and consumed ATP. The inhibitory activities were calculated on the basis of maximal activities measured in the absence of inhibitor. The IC_50_ was defined as the concentration of each compound that reduces a 50% the enzymatic activity with respect to that without inhibitors. Each value is the mean of two independent experiments.

### Calculation of physical chemical properties and CNS MPO score

Physical chemical properties of the tested compounds in the unionized form were calculated in ACDLabs PhysChem Suite 14.0^11^ – calculated logarithm of the *n*-octanol-water partition coefficient for non-ionized species (ClogP); calculated n-octanol-water distribution coefficient at pH = 7.4 (ClogD); molecular weight (*M*_W_); topological polar surface area (TPSA); number of hydrogen bond donors (HBD); logarithm of dissociation constant for the most basic centre (pK_a_) and water solubility at pH = 7.4 (ClogS_7.4_).

### Parallel artificial membrane permeability assay (PAMPA)

Prediction of the BBB penetration was evaluated using a parallel artificial membrane permeability assay (PAMPA) using the setup according to Di et al.^12^. Ten commercial drugs, phosphate buffer saline solution at pH 7.4 (PBS), Ethanol and dodecane were purchased from Sigma, Acros organics, Merck, Aldrich and Fluka. The porcine polar brain lipid (PBL) (catalogue no. 141101) was from Avanti Polar Lipids. The donor plate was a 96-well filtrate plate (Multiscreen^®^ IP Sterile Plate PDVF membrane, pore size is 0.45 µM, catalogue no. MAIPS4510) and the acceptor plate was an indented 96-well plate (Multiscreen^®^, catalogue no. MAMCS9610) both from Millipore. Filter PDVF membrane units (diameter 30 mm, pore size 0.45 µm) from Symta were used to filtered the samples. A 96-well plate UV reader (Thermo Scientific, Multiskan spectrum) was used for the UV measurements. Test compounds [(3–5 mg of caffeine, enoxacine, hydrocortisone, desipramine, ofloxacine, piroxicam, and testosterone), (12 mg of promazine) and 25 mg of verapamile and atenolol] were dissolved in EtOH (1000 µL). About 100 µL of this compound stock solution was taken and 1400 µL of EtOH and 3500 µL of PBS pH = 7.4 buffer were added to reach 30% of EtOH concentration in the experiment. These solutions were filtered. The acceptor 96-well microplate was filled with 180 µL of PBS/EtOH (70/30). The donor 96-well plate was coated with 4 µL of porcine brain lipid in dodecane (20 mg mL^−1^) and after 5 min, 180 µL of each compound solution was added. About 1–2 mg of every compound to be determined their ability to pass the brain barrier were dissolved in 1500 µL of EtOH and 3500 µL of PBS pH = 7.4 buffer, filtered and then added to the donor 96-well plate. Then the donor plate was carefully put on the acceptor plate to form a “sandwich”, which was left undisturbed for 2 h and 30 min at 25 °C. During this time the compounds diffused from the donor plate through the brain lipid membrane into the acceptor plate. After incubation, the donor plate was removed. UV plate reader determined the concentration of compounds and commercial drugs in the acceptor and the donor wells. Every sample was analysed at three to five wavelengths, in three wells and in two independent runs. Results are given as the mean [standard deviation (SD)] and the average of the two runs is reported. 10 quality control compounds (previously mentioned) of known BBB permeability were included in each experiment to validate the analysis set.

## Results and discussion

Compounds were screened against human recombinant enzyme CK1δ at a fixed concentration of 10 µM. When the inhibition found was more than 50%, IC_50_ value was determined and the most potent compounds with an IC_50_ <1 µM were further tested against CK1ε (Table 1).

**Table 1.** Tested compounds and the results of their *in vitro* evaluation on CK1δ/CK1ε inhibition together with the previously published ABAD inhibition data^7^.


Compound	*R*^1^	*R*^2^	IC_50_ CK1δ (µM**** ****±**** ****SD)	IC_50_ CK1ε (µM**** ****±**** ****SD)	SI^a^	ABAD inhibition at 25 μM (% of control**** ****±**** ****SEM)
**K684**	F	4-OH	1.37 ± 0.23	ND^b^	–	94.42 ± 1.13
**K685**	Cl	4-OH	0.59 ± 0.06	9.01 ± 0.37	15.3	91.93 ± 1.93
**K686**	F	3-OH	0.86 ± 0.10	4.02 ± 0.16	4.7	94.80 ± 0.97
**K687**	Cl	3-OH	0.16 ± 0.03	1.92 ± 0.38	12.0	93.58 ± 1.87
**K688**	F	2-OH	0.90 ± 0.14	7.33 ± 0.29	8.1	91.46 ± 1.51
**K689**	Cl	2-OH	0.48 ± 0.08	3.91 ± 0.40	8.1	77.06 ± 0.99
**K690**	F	3-Cl, 4-OH	0.84 ± 0.16	10.12 ± 0.74	12.0	39.76 ± 0.48
**K691**	Cl	3-Cl, 4-OH	0.73 ± 0.21	3.01 ± 0.33	4.1	38.61 ± 0.70
**K692**	F	3-COOH, 4-OH	0.91 ± 0.31	8.41 ± 0.71	9.2	101.30 ± 1.61
**K693**	Cl	3-COOH, 4-OH	0.40 ± 0.03	2.29 ± 0.12	5.7	100.54 ± 1.05
**K694**	F	4-OMe	7.38 ± 0.93	ND	–	110.95 ± 1.84
**K695**	Cl	4-OMe	8.87 ± 0.73	ND	–	104.55 ± 1.16
**K696**	F	3,4-OMe	>10 (23%)	ND	–	103.59 ± 1.91
**K697**	Cl	3,4-OMe	0.69 ± 0.19	>10 (26%)	>14.5	104.36 ± 1.45
**K698**	F	3-COOH, 4-OMe	3.37 ± 0.40	ND	–	105.50 ± 1.48
**K699**	Cl	3-COOH, 4-OMe	0.46 ± 0.17	3.49 ± 0.09	7.6	107.22 ± 2.54
**K700**	F	4-OPh	>10 (36%)	ND	–	108.75 ± 2.20
**K701**	Cl	4-OPh	>10 (37%)	ND	–	107.42 ± 1.93
**K702**	F	4-COOH	2.99 ± 0.36	ND	–	107.80 ± 1.75
**K703**	Cl	4-COOH	0.30 ± 0.04	3.69 ± 0.41	12.3	105.50 ± 1.68
**K704**	F	4-COOEt	>10 (31%)	ND	–	110.28 ± 1.71
**K705**	Cl	4-COOEt	>10 (45%)	ND	–	108.56 ± 2.21
**K706**	F	4-COOMe	>10 (48%)	ND	–	114.67 ± 5.35
**K707**	Cl	4-COOMe	8.73 ± 0.71	ND	–	99.31 ± 3.24
**K708**	F	4-NHCOMe	>10 (39%)	ND	–	94.70 ± 3.27
**K709**	Cl	4-NHCOMe	1.32 ± 0.15	ND	–	97.88 ± 3.40
**K710**	F	3-COOMe, 4-OH	>10 (24%)	ND	–	97.23 ± 3.84
**K711**	Cl	3-COOMe, 4-OH	9.98 ± 0.96	ND	–	95.62 ± 4.49

a Selectivity index = IC_50_CK1ε/IC_50_CK1δ.

b Not determined.

All compounds showed at least some inhibitory activity towards CK1δ (Table 1). The most potent CK1δ inhibitors were found compounds **K687**, **K689**, **K693**, **K699** and **K703** with IC_50_ values lower than 0.5 µM. Regarding CK1ε inhibition, all compounds tested showed weaker activity compared to CK1δ inhibition. Best CK1ε inhibitors **K687** and **K693** had IC_50_ values around 2 µM. The selectivity of compounds towards CK1δ inhibition was indicated using the selectivity index (SI = IC_50_ CK1ε/IC_50_ CK1δ), which showed that the inhibitors are about 10 times more active towards CK1δ.

The best inhibitor found for both CK1δ (IC_50_ = 0.16 µM) and CK1ε (IC_50_ = 1.92 µM) was compound **K687** with chlorine substitution on benzothiazolyl moiety and hydroxyl group in position 3 of the phenyl ring. Generally, substitution in position 6 of benzothiazolyl moiety with chlorine was superior to fluorine. Substitution of the phenyl ring with either hydroxyl or carboxyl group had positive effect on inhibitory activity possibly due to their ability to act as HBD and therefore allowing additional interaction with the enzyme. Only compounds **K710** and **K711** did not follow this trend being poor inhibitors. A possible explanation for this discrepancy is that an intramolecular H-bond is created between the hydroxyl and ester group resulting in non-availability of the hydroxyl proton for hydrogen bonding with the enzyme. As seen for compounds **K684**–**K689** different positioning of hydroxyl group on the phenyl ring had only limited influence on the activity. In contrast with the general SAR stated here, the dimethoxy derivative **K697** also showed good inhibitory ability, although it does not possess a hydrogen bond donor on its phenyl ring.

The best CK1δ inhibitor **K687** showed to be more potent in comparison to standards **IC261** (IC_50_ = 1 µM for both CK1δ and CK1ε) and **D4476** (IC_50_ = 0.2 µM for CK1δ), which are the most characterized selective CK1 inhibitors for *in vitro* and *in vivo* applications^1^. Still, the best inhibitors developed by Salado et al. showed even 10 times higher activity compared to **K687**, however, the increased activity was reserved only for compounds with trifluoromethyl substitution in position 6 on benzothiazolyl moiety. In direct comparison of 6-chloro and 6-flouro substituted compounds, some benzothiazolylphenylureas showed slightly increased activity compared to corresponding benzothiazolylphenylacetamide^6^. This finding leads us to assumption, that replacing of the chlorine substitution on the benzothiazole moiety of **K687** with trifluoromethyl group could further improve the inhibitory ability.

Most interestingly, compounds **K690** and **K691** showed good inhibitory activity towards both CK1δ (IC_50_ = 0.84 µM and 0.73 µM) and ABAD (39.8% and 38.6% inhibition at 10 µM^7^; IC_50_ = 1.89 µM and 1.67 µM) and such dual-activity could be of advantage for targeting AD. It is assumed that complex disorders, such as AD, could be more effectively targeted by multipotent compounds (also called multi-target directed ligands – MTDLs) able to intervene simultaneously in the different pathological events underlying the etiology of AD^10^ ^,^ ^13^.

One of the main obstacles for the treatment of the diseases of the central nervous system (CNS) is the drug’s penetration across the BBB at therapeutic concentrations. The BBB is a complex interface between blood and the central nervous system that strictly controls the exchanges between the blood and brain compartments^14^. This barrier is composed by endothelial cells with tight junctions that protect the brain from endogenous materials which could damage the brain tissues^15^. The majority of CNS drugs enter the brain by transcellular passive diffusion, due to the tight junction structure and limited transport pathways. Thus, we have calculated the physical chemical properties^11^ of the tested compounds and used the CNS multiparametre optimization (MPO) developed by Wager et al.^16^ to predict and compare the likeliness of BBB-permeation. The MPO scoring function is based on six fundamental physical chemical parameters commonly used by medicinal chemists – calculated partition coefficient (ClogP); calculated distribution coefficient at pH = 7.4 (ClogD); molecular weight (*M*_W_); TPSA; number of HBD; most basic centre (p*K*_a_). All properties are weighted equally, with a desirability score ranging from 0.0 to 1.0 for and therefore a total CNS MPO desirability score ranges from 0.0 to 6.0. The MPO score was found in optimal range (≥4) for about half of the compounds (**K684**–**K686**, **K688**–**K689**, **K694**–**K698**, **K702**, **K706**, **K708**–**K709**; Table 2), suggesting that these compounds are likely to permeate through BBB. Looking at the separate physical chemical parameters, the TPSA (TPSA = 40–90) and solubility (ClogS_7.4_ ≥ (−3)) were the most often violated predictors (Table 2), which should be taken into account when designing the follow-up series of CK1 resp. ABAD inhibitors. Although solubility is not included in the MPO scoring function, it is crucial for the prospective biological evaluation which cannot be properly performed without reaching the desired concentrations of compounds in aqueous solutions.

**Table 2.** Calculated physical chemical properties and MPO scoring for the urea compounds^11^ ^,^ ^16^.

	PhysChemValues							
Compound	ClogP	ClogD_7.4_	TPSA	*M*_W_	HBD	p*K*_a_	ClogS_7.4_	MPO
Optimal properties	1–5	0–3	≤ (60–70)	≤400	≤3	4–10	≥ (−3)	4–6
**K684**	2.80	2.43	102.49	303.31	3	8.18	−4.2	**4.4**
**K685**	3.40	2.92	102.49	319.77	3	8.00	−4.4	**4.1**
**K686**	2.90	2.52	102.49	303.31	3	8.00	−3.7	**4.5**
**K687**	3.60	3.11	102.49	319.77	3	7.82	−4.2	**3.9**
**K688**	2.91	2.23	102.49	303.31	3	7.86	−3.4	**4.6**
**K689**	3.50	2.68	102.49	319.77	3	7.68	−4.2	**4.2**
**K690**	3.77	3.12	102.49	337.76	3	7.69	−4.6	**3.8**
**K691**	4.33	3.54	102.49	354.21	3	7.51	−4.9	**3.3**
**K692**	3.39	−0.07	139.79	347.32	4	3.07	−1.1	**3.8**
**K693**	3.90	0.36	139.79	363.78	4	3.07	−1.6	**3.5**
**K694**	3.21	2.84	91.49	317.34	2	8.05	−4.8	**4.9**
**K695**	3.89	3.42	91.49	333.79	2	7.87	−5.0	**4.3**
**K696**	3.08	2.61	100.72	347.36	2	7.88	−4.5	**4.8**
**K697**	3.71	3.12	100.72	363.82	2	7.71	−4.6	**4.2**
**K698**	3.23	−0.05	128.79	361.34	3	4.01	−1.7	**4.0**
**K699**	3.63	0.26	128.79	377.80	3	4.01	−1.9	**3.7**
**K700**	4.85	4.46	91.49	379.41	2	8.01	−5.3	**3.4**
**K701**	5.44	4.94	91.49	395.86	2	7.83	−5.8	**3.2**
**K702**	3.36	0.42	119.56	331.32	3	4.29	−1.8	**4.0**
**K703**	3.93	0.89	119.56	347.78	3	4.29	−2.4	**3.7**
**K704**	3.83	3.78	108.56	359.37	2	7.77	−5.3	**3.6**
**K705**	4.50	4.42	108.56	375.83	2	7.59	−5.9	**3.0**
**K706**	3.17	3.11	99.33	329.35	2	7.72	−4.9	**4.6**
**K707**	3.80	3.72	99.33	345.80	2	7.54	−5.7	**3.9**
**K708**	2.45	2.22	111.36	344.36	3	8.01	−4.3	**4.3**
**K709**	3.05	2.74	111.36	360.82	3	7.84	−4.9	**4.1**
**K710**	3.73	3.26	128.79	361.35	3	7.92	−4.3	**3.2**
**K711**	4.29	3.70	128.79	377.80	3	7.74	−4.7	**2.5**

Detailed view at physical chemical properties of the most promising compounds **K690** and **K691** (dual inhibitors of CK1 and ABAD) revealed that both compounds do not fully meet the criteria for BBB-penetration set by the CNS–MPO model^16^ showing overall score 3.8 resp. 3.3 (optimal range = 4–6). Both compounds exceeded the optimal value of ClogP (≤3), ClogD (≤2), TPSA (40–90) and HBD (≤0.5) with compound **K690** having slightly better results in the first two parameters. Still, both compounds fit into the less strict criteria of “tolerable” values in the four above mentioned parameters. The remaining two parameters *M*_W_ (≤350) and p*K*_a_ (≤8) fully met the CNS-MPO criteria.

**Figure 3.**
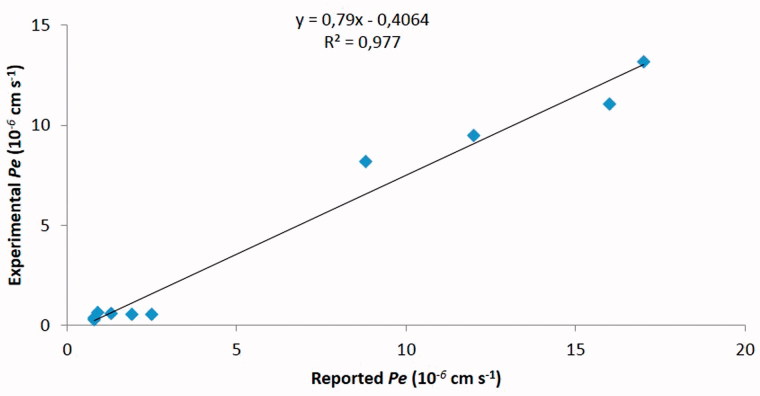
Linear correlation between experimental and reported permeability of commercial drugs using the PAMPA–BBB assay.

Parallel Artificial Membrane Permeability Assay (PAMPA) is technique developed to predict passive permeability through biological membranes. In order to further explore the capacity of compounds **K690** and **K691** to penetrate into the brain, we used the PAMPA–BBB method described by Di et al.^12^, which employed a brain lipid porcine membrane. The *in vitro* permeability (*Pe*) of commercial drugs through lipid membrane extract together with compounds **K690** and **K691** were determined and described in Table 3. An assay validation was made comparing the reported permeability values of commercial drugs with the experimental data obtained employing this methodology. A good correlation between experimental-described values was obtained *Pe* (exptl) = 0.79 (bibl) – 0.4064 (*R* ^2^ = 0.977) (Figure 3). From this equation and following the pattern established in the literature for BBB permeation prediction^17^ we could classify compounds as CNS + when they present a permeability >2.75 × 10^−6 ^cm s^−1^. Based on these results we can consider that compounds **K690** and **K691** are able to cross the BBB by passive permeation (Table 3), although their calculated physical chemical properties do not fully meet the criteria suggested by CNS-MPO model (Table 2).

**Table 3.** Permeability (*Pe* 10^−6 ^cm s^−1^) in the PAMPA-BBB assay for 10 commercial drugs (used in the experiment validation) and compounds **K690** and **K691** with their predictive penetration in the CNS^a^.

Compound	Bibl.^12^	*Pe* (10^−6^ cm s^−1^)^b^	Prediction
atenolol	0.8	0.3 ± 0.1	
caffeine	1.3	0.6 ± 0.1	
desipramine	12	9.5 ± 0.9	
enoxacin	0.9	0.6 ± 0.1	
hydrocortisone	1.9	0.5 ± 0.4	
ofloxacin	0.8	0.4 ± 0.1	
piroxicam	2.5	0.5 ± 0.1	
promazine	8.8	8.1 ± 0.1	
testosterone	17	13.9 ± 1.9	
verapamil	16	11.0 ± 1.2	
**K690**		13.2 ± 1.4	CNS+
**K691**		14 ± 2	CNS+

a PBS:EtOH (70:30) was used as solvent.

b Data are the mean ± SD of 2 independent experiments.

## Conclusions

Based on structural similarity with known CK1 inhibitors, 28 compounds originally designed as ABAD inhibitors were evaluated for their inhibitory activity on CK1 and their potential to cross the BBB was predicted using CNS-MPO model and eventually PAMPA. Several novel CK1 inhibitors with IC_50_ in high nanomolar to low micromolar range were identified with compound **K687** being the best hit (IC_50_ = 0.16 µM/1.92 µM for CK1δ resp. CK1ε). Moreover, compounds **K690** and **K691** were shown to be low micromolar inhibitors of both, CK1 and ABAD, and hence they present a potential novel class of dual-acting anti-AD therapeutics. The results of PAMPA for **K690** and **K691** suggests that the compounds should be able to penetrate into the brain.
